# Response to “Commentary on picosecond neodymium-doped yttrium aluminum garnet laser therapy for pigmentation due to lichen planus pigmentosus in a patient with skin of color”

**DOI:** 10.1016/j.jdcr.2023.08.029

**Published:** 2023-09-01

**Authors:** Kathleen C. Suozzi

**Affiliations:** Department of Dermatology, Yale School of Medicine, New Haven, Connecticut

*To the Editor:* Thank you for your response to this single case report highlighting successful treatment of hyperpigmentation due to lichen planus pigmentosus. As you mention, this is an “orphan disease” and we are glad this report has stimulated a discussion as that was the intent.

We appreciate you pointing out a typo that will be clarified here: Line 54: “Q-second” is not a proposed new term and should have read “Q-switched.” While nanosecond laser is a more specific description of the pulse duration of these lasers, this laser class is characterized by the use of a flashlamp pumped solid-state resonator in combination with an optical modulator or Pockel’s cell to generate output in the nanosecond domain. While such pulse generation is most accurately described as *actively* Q-switched, it is regularly referred to in the medical literature simply as Q-switched. We to want to point out that the picosecond laser used in this report (PicoWay, Candela) is a flashlamp pumped solid-state laser whose output is simultaneously injected into both an ultrashort master oscillator called a seed laser, which is *passively* Q-switched, and into an ultrafast optical Power Amplifier. Arranged in a classical MOPA configuration, the PicoWay generates pulses with high peak-powers in the picosecond domain. While it leverages a passively Q-switched seed laser internally, the more important characteristic is its pulse width, and it is most often referred to in the medical literature as a picosecond laser.

To clarify the energy settings, a range of settings were used using both the 1064 nm and 785 nm handpiece. After trials of lower fluence settings were ineffective, the treatments were titrated up to 2 to 2.4 J/cm^2^ with 1064 nm at a 3 to 4 mm spot size and 0.6 to 1.5 J/cm^2^ with 785 nm and a 3 to 4 mm spot size. You are correct that these energies are higher than are recommended for treating other disorders of pigmentation, such as melasma or post-inflammatory hyperpigmentation from other causes. It is because of our findings at these settings that we believe the case was valuable to report. When treating hyperpigmentation in skin of color, one needs to respect endogenous melanin. This can be particularly challenging when the pigmentation demarcation is ill-defined or patch-like. In these patients, it is this author's opinion that low fluence settings should be employed to minimize risk of dyspigmentation. The patient reported in this case was unique in that the areas of hyperpigmentation were punctate macules coalescing in some areas to larger well-defined patches. Because of the discrete nature of the hyperpigmentation in this patient’s presentation, we believe higher fluences were able to be used successfully. Due to editorial constraints we included only the settings that were ultimately efficacious for the patient and not the trial settings which were more conservative and not effective. As Dr Wambier points out, clinical end points of laser treatment are critical to define and can be more challenging to assess in skin of color. We agree with his assessment that frosting would be too aggressive an end point. The end point in this patient was mild erythema.

The goal of this publication is to highlight that picosecond lasers can be considered for treatment of hyperpigmentation secondary to lichen planus pigmentosus in skin of color. We are not suggesting that they are a treatment modality that should be used widely and indiscriminately. Ultimately, a larger trial would best assess a variety of types of lichen planus pigmentosus and determine appropriate treatment settings. We look forward to the data you will soon be reporting.

## Conflicts of interest

None disclosed.

